# *Notes from the Field:* Monitoring Out-of-State Patients During a Hurricane Response Using Syndromic Surveillance — Tennessee, 2017

**DOI:** 10.15585/mmwr.mm6649a6

**Published:** 2017-12-15

**Authors:** Caleb Wiedeman, Julie Shaffner, Kelly Squires, Jeffrey Leegon, Rendi Murphree, Paul E. Petersen

**Affiliations:** ^1^Tennessee Department of Health; ^2^Division of State and Local Readiness, Office of Public Health Preparedness and Response, CDC.

In late August and early September of 2017, Hurricanes Harvey and Irma swept through the Caribbean and made landfall in the continental United States. As Texas and Florida readied for direct impacts of the storms, nearby states prepared for the arrival of internally displaced persons. During the weeks surrounding the storms, the Tennessee Department of Health (TDH) supported all-hazards situational awareness for public health partners by enhancing syndromic surveillance activities, i.e., the monitoring of symptom combinations or other indicators within a population to inform public health action (*1*).

TDH collects and analyzes emergency department (ED) data from 70 hospitals across Tennessee using the Electronic Surveillance System for Early Notification of Community-Based Epidemics (ESSENCE) (*2*). ESSENCE is a tool in the BioSense Platform made available to public health jurisdictions through CDC’s National Syndromic Surveillance Program (NSSP) (*3*). Syndromic surveillance typically supplements disease- or condition-specific surveillance; however, it can also improve situational awareness during an event or disaster.

Before, during, and after the landfalls of Hurricanes Harvey and Irma, the volume of out-of-state patients visiting EDs in Tennessee was monitored to identify any unusual clusters of symptoms or spatial clustering and to assess the real-time impact on the health care system by persons displaced by the storms.

Data were monitored from August 18–September 24, 2017 by querying ESSENCE for patient home postal codes in Texas and Florida. During the monitoring period, Tennessee EDs reported 257,095 total visits, including 277 (0.1%) patient visits by Texas residents and 1,041 (0.4%) visits by Florida residents. The number of ED visits by patients from Texas remained stable during the monitoring period (average 7.3 per day). In contrast, there was an increase in patients from Florida visiting Tennessee EDs beginning 3 days before Hurricane Irma made landfall in the continental United States. The increase peaked on the day of impact in Florida (September 10) at 116 ED visits, and returned to baseline levels (between 10–20 patients per day) within 1 week (Figure). The increase in patients from Florida was evenly distributed across Tennessee, with some clustering around a popular tourism area in East Tennessee. No concerning trends in reported syndromes or chief complaints were identified among Texas or Florida patients. The most frequently occurring chief complaints for these patients were injuries, complaints of chest or back pain, gastrointestinal illness, and respiratory illness, similar to patterns of chief complaints seen in Tennessee residents visiting EDs during the same period.

**FIGURE:**
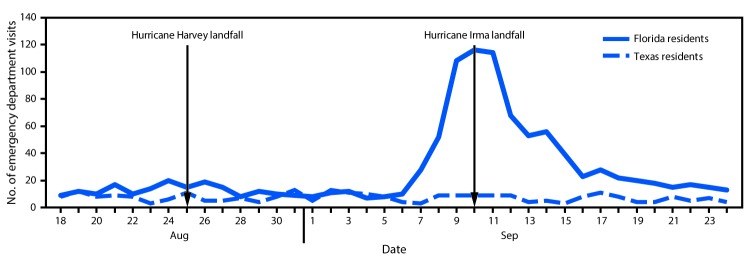
Emergency department visits* by residents of Texas and Florida — Tennessee, August 18, 2017–September 24, 2017 *Identified through query of the Electronic Surveillance System for Early Notification of Community-Based Epidemics (ESSENCE).

Syndromic surveillance data are often used to identify clusters of illness based on geography or time (*1*); TDH was able to use the data to detect changes suggestive of population displacement due to an out of state natural disaster. Although TDH was unable to validate whether patients identified as residents of Florida were displaced because of Hurricane Irma, the timing of the increase and subsequent decrease in patient ED visits suggested population displacement related to the storm. The absence of a substantial increase in patients with residence in Texas suggested that the effects of Hurricane Harvey were not affecting hospital EDs in Tennessee.

At TDH, ESSENCE is the only easily accessible information source capable of rapidly collecting health information on out-of-state patients. This initiative allowed TDH to observe where and when out-of-state patients were seeking ED care in Tennessee and to monitor the need for targeted messaging and resources to heavily affected areas. Additionally, close surveillance of chief complaints among out-of-state patients provided assurance that no unusual patterns in illness or injury were occurring.

Enhancing syndromic surveillance during these storms was an important strategy for improving situational awareness among public health stakeholders and will be incorporated into future response activities by TDH.
